# The Quality of Parent-Adolescent and Peer-Adolescent Relationships and Trust in Strangers

**DOI:** 10.1177/02724316251379489

**Published:** 2025-09-23

**Authors:** Hester Sijtsma, Mariët van Buuren, Jacek Buczny, Miriam Hollarek, Reubs J. Walsh, Nikki C. Lee, Lydia Krabbendam

**Affiliations:** 1 Department of Clinical, Neuro- and Developmental Psychology, Faculty of Behavioral and Movement Sciences, 100432 Vrije Universiteit Amsterdam, Research Institute LEARN!, Institute for Brain and Behavior Amsterdam, Amsterdam, Netherlands; 2 Department of Experimental and Applied Psychology, Faculty of Behavioural and Movement Sciences, Vrije Universiteit Amsterdam, Amsterdam, Netherlands

**Keywords:** trust behavior, parent-adolescent relationship, peer-adolescent relationship, adolescence

## Abstract

Trust experiences gained from close others may generalize to trust interactions with strangers. In this longitudinal study, we examined the association between the quality of relationships with parents and peers and trust in strangers over age. The three waves were 12 months apart (parent relationship: *N*: 547, *M*_age_: 12.5; *N*: 505, *M*_age_: 13.5; *N*: 406, *M*_age_: 14.5; peer relationship: *N*: 567, *M*_age_: 12.6; *N*: 511, *M*_age_: 13.5; *N*: 416, *M*_age_: 14.5). The quality of parent and peer relationships were measured using the Inventory of Parent and Peer Attachment-Revised. Trust was measured using an experimental paradigm called the Trust Game. Using multi-level analyses, no evidence was found for age-related changes across the three waves regarding the association between the quality of the parent-adolescent and the peer-adolescent relationship, and trust. Unexpectedly, the results showed weak evidence for a negative relationship between the quality of parent-adolescent relationships and trust in strangers independent of age. Given the non-significant findings, we recommend future studies to differentiate between relationships with different caregivers.

## Introduction

Trust plays a crucial role in social functioning. Trusting others is essential in relationships with close others, such as with family members and friends. However, trust extends beyond close relationships to trust in strangers, for example, during interpersonal interactions such as encounters on the street or on the train. Trust is also important for the cohesion and success of societies, as it plays a key role in democracy, economy, and politics (Stolle, 2002), highlighting the importance that individuals trust each other to behave in conventional ways (Herreros, 2023; Putnam, 2000; Uslaner, 2018). Besides the beneficial effects that high levels of trust in strangers may have for society, a basic sense of trust in strangers is assumed to be crucial for healthy psychosocial development (Erikson, 1963; Rotenberg et al., 2023; Schilke et al., 2021; Terrell et al., 2000).

Trust may become particularly important during adolescence, as this is a time of life when young people expand their social world and start to occupy a position in and contribute to society (Brown & Larson, 2009; Erdley & Day, 2017). New people are met, specifically outside the family environment, and social interactions with strangers become more frequent. Studies have presented mixed findings regarding whether trust in strangers increases throughout adolescence (cross-sectional studies: Sutter & Kocher, 2007; Van de Groep et al., 2018; Van den Bos et al., 2012); however, a recent longitudinal study showed an age-related increase in trust in strangers from ages 11 to 16 (Sijtsma et al., 2023). Trust interactions with strangers might be influenced by the interactions and quality of relationships one has with close others (Stolle, 2002). For adolescents, this means that the experience of high-quality relationships with parents and friends may generalize to how trust interactions with strangers are approached (Feeney et al., 2008). In the current study, we aim to better understand how adolescent relationships with close others are associated with trust in strangers throughout adolescence, as this type of trust plays an important role on interpersonal as well as societal levels.

Attachment theory posits that individuals possess working models based on experiences with close others (for example, parents and friends) that represent core aspects of the world of the individual and their attachment figures, such as the attachment figure’s accessibility, availability, and responsivity (Bowlby, 1969, 1973, 1980). These working models grow throughout development and are used to forecast and generalize to new social situations. Based on the attachment theory, one can reason that the parent-child relationship and the interactions between parents and children serve as a foundation for how trust interactions with strangers are approached (also see Feeney et al. (2008) and Stolle (2002)). Although some parents may teach their children not to trust strangers (Stolle & Nishikawa, 2011), trust is a public good (Stolle, 2002), and as all close relationships start off with being strangers to one another, interactions with strangers are suggested to reflect individuals’ working models (Feeney et al., 2008). As such, previous studies have linked parental attachment to social-cognitive behaviors that people appeal to during social interactions with strangers. Securely-attached adolescents were found to show greater support-seeking and support-giving behaviors to strangers (Feeney et al., 2008), while adolescents with high avoidance attachment showed less empathic ability during interactions with strangers (Izhaki‐Costi & Schul, 2011).

This generalization effect on how the relationship and experiences with close others can transfer to situations with strangers may become especially relevant during adolescence. The parent-child relationship reorganizes as adolescents gain more autonomy and independence, shifting their attention from the family environment to their peers (Branje, 2018; Smetana & Rote, 2019). The adolescents’ need for autonomy can potentially elicit moments of conflict and turbulence, leading to a decrease in the quality of the parent-adolescent relationship, which is observed more often during early adolescence than late adolescence (Branje, 2018; Smetana & Rote, 2019). As such, it is probable that the proposed association between the quality of the parent-child relationship and trust in strangers becomes weaker during adolescence.

At the same time, peer relationships and friendships become increasingly important during adolescence (Burnett Heyes et al., 2015; Meeus, 2016). New people are met, and the quality of friendships increases (Van Harmelen et al., 2021), especially for girls (Delgado et al., 2022), and adolescents start to occupy a position in and contribute to society (Fuligni, 2019). The increased importance of peers and friends during adolescence may mean that the proposed association between the quality of the peer-adolescent relationship and trust in strangers becomes stronger throughout adolescence. Rotenberg and Boulton (2013) and Rotenberg et al. (2004) showed that already during childhood general trust beliefs in others are strengthened through repeated interactions with known peers. Also, children showed that having positive peer relationships was related to trusting friend and non-friend classmates and having general trust beliefs in others (Chin, 2014; Jambon & Malti, 2022), while negative peer experiences promoted a negative (i.e., untrustworthy) view of others (Dodge, 2006). This work indicates that positive peer interactions may boost children’s working model on how they view unknown others (Bernath & Feshbach, 1995; Rotenberg & Boulton, 2013). This may even further evolve during adolescence when social life expands tremendously and friends become increasingly important.

There has been limited empirical work on the association between the quality of relationships with parents and peers and trust in strangers in adolescents. A recent study showed that, in adolescents, the level of embeddedness within friendship networks was related to trust behavior in strangers (Sijtsma et al., 2023). One study in adults showed that the level of feeling connected to peers in one’s personal social network was positively associated with the level of trust in unknown people (Welch et al., 2007). In sum, whether experiences gained from relationships with close others generalize to trust in strangers has been examined to a limited extent. It is interesting to explore the generalization effect in more detail, especially in a sample of adolescents as they are in a period of life when social relationships change, new people are met, and opportunities to contribute within the broader community emerge.

In the current study, we examined the association between the quality of relationships with significant others (that is, parents and friends) and trust in strangers from early to mid-adolescence. The current study’s research aim, hypotheses, and planned analyses were preregistered on Open Science Framework (http://osf.io/br3p9). As reported in the preregistration, the Trust Game data examined in the current study was also analyzed in an earlier publication by our group to study the development of adolescent trust behavior (Sijtsma et al., 2023). The results of this earlier study showed a significant age-related increase in trust behavior in strangers and that this increase was stronger for boys than for girls.

In the current study, we first examined developmental effects for which we expected the association between the quality of parent-adolescent relationships and the level of trust in strangers to decrease as adolescents became older because the autonomy and independence from parents increases throughout adolescence (Hypothesis 1: the strength of the association between the quality of parent-adolescent relationships and trust behavior becomes weaker with age). At the same time, peer relationships (i.e., friends) become more important, so we expected the association between the quality of peer-adolescent relationships and the level of trust in strangers to increase with age (Hypothesis 2: the strength of the association between the peer-adolescent relationships and trust behavior becomes stronger with age). Independent of age, we expected that higher-quality relationships with parents were related to higher levels of trust behavior (Hypothesis 3) and that the quality of peer-adolescent relationships and trust in strangers were positively associated as well (Hypothesis 4). Gender differences were also explored but we did not have specific expectations regarding the direction of the group differences, because prior studies show contrasting results regarding gender differences and social-cognitive functions implicated in trust behavior (Białecka-Pikul et al., 2021; Boele et al., 2019; Jambon & Malti, 2022).

## Method

### Participants

The current study is part of the longitudinal #SOCONNeCT project that involved six waves of data collection (also see Sijtsma et al., (2021)). Data was collected at eight secondary schools in the Netherlands. The participants were enrolled in the general secondary educational track or the pre-university educational track, which form the higher levels of education within the Dutch education system (these two higher levels constitute the top 40% of pupils based on academic achievement). The Trust Game and the Inventory of Parent and Peer Attachment-Revised (IPPA-R) were collected during Wave 1, Wave 3, and Wave 5 of the #SOCONNeCT project (and not during the remaining waves). There was a 12-month interval between the waves. Wave 1 took place at the beginning of the first year of secondary school, Wave 3 at the beginning of the second year, and wave 5 at the beginning of the third year of school. Schools received €7.50 per participating pupil per wave. The financial compensation was intended to be used for class activities. An additional payout could be earned based on a participant’s average earnings per trial in the Trust Game and the average payouts were added to the financial compensation.

**Figure 1.**
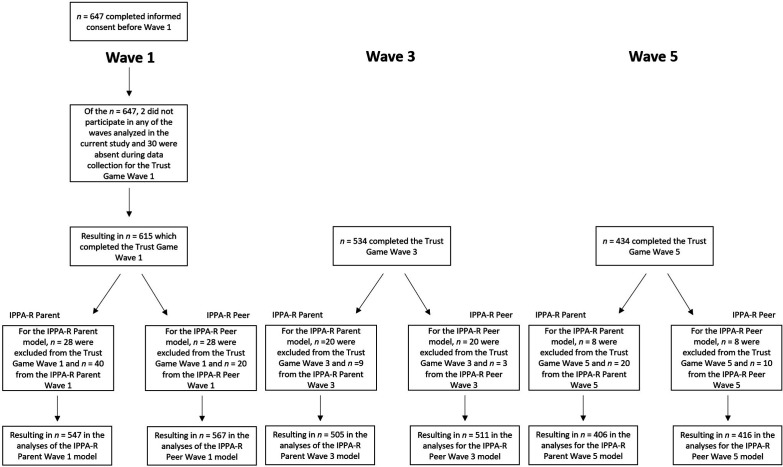
The Flow Chart of the Inclusion Process of the Participants in the Current Study

In total, 647 adolescents provided written informed consent for participation in the #SOCONNeCT project. Of these participants, 615 completed the Trust Game during Wave 1 (see Figure 1). After data exclusion (see Figure 1), 547 participants were included in the analyses of the IPPA-R Parent Wave 1 model (*M*_age_ = 12.5 years, *SD*_age_ = 0.4 years, range: 11.1-14.0 years, 253 boys) and 567 participants in the analyses of the IPPA-R Peer Wave 1 model (*M*_age_ = 12.6 years, *SD*_age_ = 0.4 years, range: 11.1-14.0 years, 263 boys). During Wave 3, 534 participants completed the Trust Game. After data exclusion (see Figure 1), 505 participants were included in the analyses of the IPPA-R Parent Wave 3 model (*M*_age_ = 13.5 years, *SD*_age_ = 0.4 years, range: 12.1-15.0 years, 236 boys) and 511 participants in the analyses of the IPPA-R Peer Wave 3 model (*M*_age_ = 13.5 years, *SD*_age_ = 0.4 years, range: 12.1-15.0 years, 240 boys). During Wave 5, 434 participants completed the Trust Game. After data exclusion (see Figure 1), 406 participants were included in the analyses of the IPPA-R Parent Wave 5 model (*M*_age_ = 14.5 years, *SD*_age_ = 0.4 years, range: 13.3-16.0 years, 184 boys) and 416 participants in the analyses of the IPPA-R Peer Wave 5 model (*M*_age_ = 14.5 years, *SD*_age_ = 0.4 years, range: 13.3-16.0 years, 189 boys).

In total, 311 participants completed the Trust Game and the IPPA-R Parent questionnaire during all three waves, 205 participants during two waves, and 115 participants during one of the waves. Furthermore, 332 participants completed the Trust Game and the IPPA-R Peer questionnaire during all three waves, 191 participants during two waves, and 116 participants during one of the waves. Of the total sample included, 99% of the participants included were born in Western European countries, while few of the participants were born in other countries, such as Asian countries, North or South American countries. Furthermore, a proxy for socio-economic status indicated that the majority of participants in the current sample resided in neighborhoods with generally above-median incomes.

### Procedure

Multiple schools throughout the Netherlands were contacted to participate in the #SOCONNeCT project. If schools were willing to participate, all pupils and parent(s)/caregiver(s) in a given class were informed about the aims and the procedures around participation in the #SOCONNeCT project through information letters and information evenings. There were no exclusion criteria for participation (the only requirement was that pupils were proficient in Dutch). Participation was discussed between pupils and their parent(s)/caregiver(s), and in case of participation, both the pupils and parent(s)/caregiver(s) provided active written informed consent prior to the start of the data collection. The data collection was conducted at school in the participants’ classrooms under the supervision of the researchers and trained research assistants and lasted around 90 min, including explanations and the administration of tasks and questionnaires that are not analyzed in the current study. Before the administration of the Trust Game, a joint explanation in class was provided on how the game works. Also, a comprehension check was completed individually (by answering three questions correctly) to check if the participants understood the procedure of the game. The Trust Game and IPPA-R were completed individually. The Trust Game was administered on a laptop, and the IPPA-R was administered on an iPad, both provided by the research team. The #SOCONNeCT project was approved by the scientific and ethical review board of the Faculty of Behavioural and Movement Sciences of the Vrije Universiteit Amsterdam.

## Materials

### Trust Game

**Figure 2.**
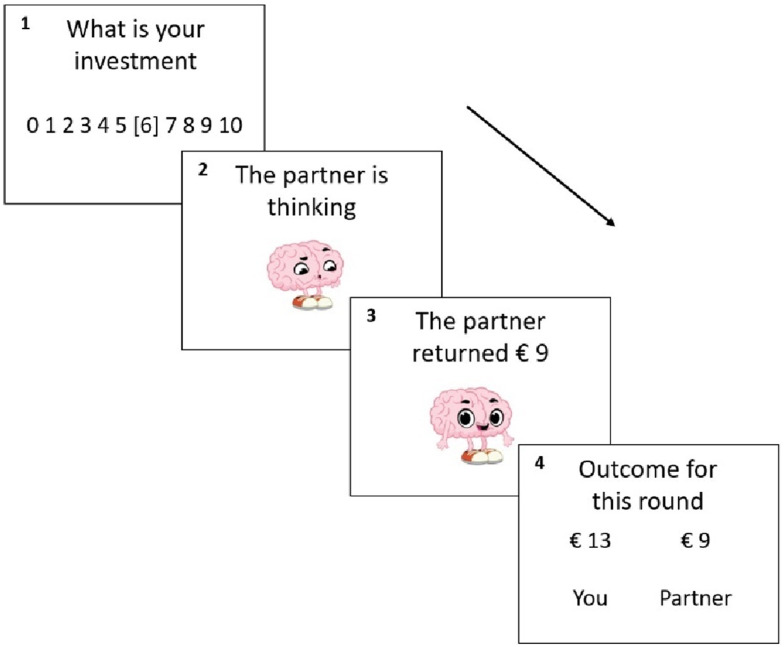
An Example of a Trial in the Trust Game *Note*. This is an example of a trial in the trustworthy condition. The investment is €6, which is multiplied by 3. This means the partner received €18, and the participant kept €4. In this example, the factor that determines the partner’s return was 1.5, meaning that the partner’s return is €9. Thus, the outcome of this round is €13 for the participant and €9 for the partner.

The Trust Game was used to measure trust behavior (Berg et al., 1995). This paradigm is regularly used in adolescent samples (e.g., Fett et al., 2014; Krabbendam et al., 2024; Lee et al., 2016; Van den Bos et al., 2010; Van den Bos et al., 2011; Van den Bos et al., 2012). The Trust Game included multiple trials. One trial consisted of four screens (see Figure 2). The first screen displayed the monetary units 0 to 10. The participants were asked to select the amount of money they wished to invest by using the arrow keys (called ‘the investment’, see Screen 1 in Figure 2). The investment was multiplied by three and received by the partner. The second screen presented a picture of the partner (i.e., a cartoon animation) accompanied by the text “The partner is thinking” (see Screen 2 in Figure 2). This was followed by a screen that revealed the partner’s return (see Screen 3 in Figure 2). The return of the partner in each trial was determined by multiplying the investment by a predefined factor using a preprogrammed algorithm. The fourth screen showed the total earnings for both players for that trial (see Screen 4 in Figure 2). See the Supplementary materials for a detailed explanation about the preprogrammed algorithm.

The Trust Game was administered during Wave 1, Wave 3, and Wave 5 of the #SOCONNeCT project. During each data collection wave, the participants completed two conditions of the game (the trustworthy condition and the untrustworthy condition) which were administered in counterbalanced order. Both conditions consisted of 15 trials. To have a measure of trust toward strangers where participants had no information or impression on the partner yet, trust was measured using the investment that the participant made during the first trial of the first condition that was administered (ranging from 0 to 10). Higher investments indicated more trust. The remaining 14 trials of the first condition and the trials of the second condition were not included in the analyses. Participants were not informed about the partner’s trustworthiness level.

As we used a longitudinal design with three waves of data collection (where debriefing would not have been possible until the final wave), it was unfeasible and undesirable to use a real human counterpart or to use deception and let participants believe that they were playing with a human counterpart while in fact they were playing with a computerized partner. Therefore, the interaction partner in the Trust Game was a cartoon character. Before the start of the game, the participants were informed that they would play two games with computer counterparts. The same Trust Game was used during each wave, but the appearances of the cartoon characters used as interaction partners varied, and they were given different names each wave to prevent potential learning effects.

### Inventory of Parent and Peer Attachment-Revised (IPPA-R)

The quality of the parent-adolescent and the peer-adolescent relationship was examined by using the IPPA-R (Greenberg & Armsden, 2009; Gullone & Robinson, 2005). The IPPA-R consists of 25 items examining the parent-adolescent relationship (IPPA-R Parent) and 25 items examining the peer-adolescent relationship (IPPA-R Peer). There were three subscales for both the IPPA-R Parent and IPPA-R Peer: the Trust subscale, the Communication subscale, and the Alienation subscale. The Trust subscale consisted of 10 items, e.g., ‘I trust my parents.’ for the IPPA-R Parent and ‘I feel my friends are good friends.’ for the IPPA-R Peer. The Communication subscale consisted of nine items, e.g., ‘If my parents know something is bothering me, they ask me about it.’ for the IPPA-R Parent and ‘When we discuss things, my friends care about my point of view.’ for the IPPA-R Peer. The Alienation subscale consisted of six items, e.g., ‘I get upset easily around my parents.’ for the IPPA-R Parent and ‘My friends don’t understand what I’m going through these days.’ for the IPPA-R Peer. The IPPA-R was administered during Wave 1, Wave 3, and Wave 5 of the #SOCONNeCT project.

Participants were instructed that they would receive questions about their parents (no distinction was made between fathers and mothers), their friends, and themselves, and that they should indicate to what extent the questions applied to them. Items were answered on a 5-point scale ranging from 1 = ‘Almost never or never true’ to 5 = ‘Almost always or always true’. Two items of the Trust subscale and two items of the Communication subscale were reverse-scored. Following the IPPA-R manual (Armsden & Greenberg, 1987), the quality of the parent-adolescent relationship and peer-adolescent relationship was calculated by adding the sum score of the Trust and Communication subscales, from which the Alienation subscale sum score was subtracted. For both types of relationships applies that higher scores indicated a higher quality relationship (Armsden & Greenberg, 1987) (see Gullone & Robinson, 2005; McMahon et al., 2020 for a similar approach). Scores could range from −11 to 89. The IPPA-R items were translated into Dutch by a bilingual native Dutch/English speaker, followed by back translations and a discussion of uncertainties with a focus group of adolescents. In the current sample, the reported Cronbach’s alpha value of the IPPA-R Parent was .67 for Wave 1, .77 for Wave 3, and .78 for Wave 5. For the IPPA-R Peer, this was .82 for Wave 1, .79 for Wave 3, and .78 for Wave 5.

### Age

Information about participants’ age was collected during each wave of data collection. Age was a continuous variable and centered for the analyses.

### Gender

Participants’ gender (boy, girl, other) was collected during Wave 1. Gender was used as a categorical variable with two levels (boy, girl) as none of the participants filled in the option ‘other’.

### Country of Birth

Information on participants’ country of birth was collected during the first wave of data collection. This information was used to describe the characteristics of the sample (see ‘Participants’).

### Proxy of Socio-Economic Status

During Wave 1, participants reported the postal code of their home address. We requested data from the Central Agency for Statistics on the average yearly gross income per postal code in the Netherlands so we could calculate a proxy for socio-economic status (CBS, 2018). This proxy was used to describe the characteristics of the sample (see ‘Participants’).

### Data Analysis

Two multilevel models were tested to examine the research aims of the current study. Each model separately examined the association between either the parent-adolescent relationship or the peer-adolescent relationship and trust behavior over age. Gender (collected during Wave 1) and the repeated measures (collected during Wave 1, Wave 3, and Wave 5) of age, and the quality of either the parent-adolescent relationship or the peer-adolescent relationship were the predictor variables. The outcome variable was the repeated measure of trust behavior (collected during Wave 1, Wave 3, Wave 5). The two model-building procedures for the parent-adolescent relationship and the peer-adolescent relationship had the same consecutive steps outlined below. Only when the model fit improved due to adding a fixed or random effect, the effect was kept in the model at the consecutive step. Model fit comparisons were done using the likelihood ratio test. Analyses were done in R version 4.3.1 (RCoreTeam, 2020). The packages *lme4* and *lmerTest* were used to perform the multilevel analyses (Bates et al., 2015; Kuznetsova et al., 2017). An advantage of using multilevel analysis is that this technique allows the inclusion of participants who participated in only one or two of the three waves and thus models can be estimated with imbalanced group sizes per wave.

As reported in the preregistration, an earlier publication by our group used the same Trust Game data as examined in the current study to study the development of adolescent trust behavior (Sijtsma et al., 2023). Consequently, the age- and gender-related results for the current dataset are known and thus the first step (Model 1) in the model-building procedure was adding the fixed main effect of age and gender. In the second step (Model 2), the random slope of age was added. In the third step (Model 3), the fixed main effect of relationship (either parent or peer) was added. In the fourth step (Model 4), the random slope of relationship was added (either parent or peer). In the fifth step (Model 5), the two-way interaction between age and relationship (either parent or peer) and the related main effects were added. Finally, in step 6 (Model 6), to exploratory test for gender differences, the three-way interaction between age, relationship (either parent or peer), and gender was added (and also the related main effects and the lower-order interactions between age and relationship and between gender and relationship).

## Results

### The Quality of the Parent-Adolescent Relationship

**Figure 3.**
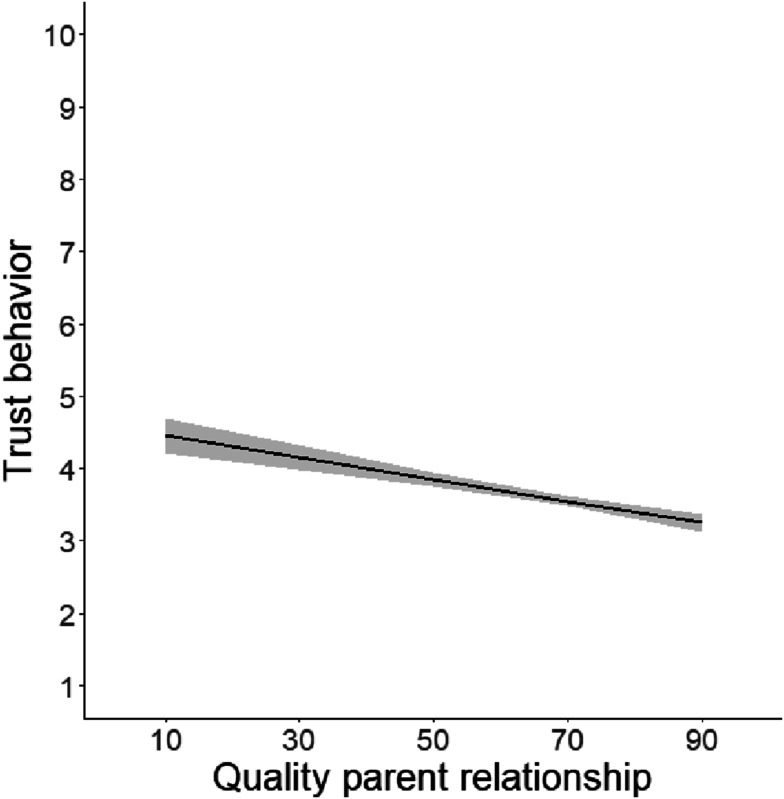
The Negative Association Between the Quality of the Parent-Adolescent Relationship and Trust Behavior

The model-building procedure that concerned the association between the parent-adolescent relationship and trust showed that the final model (i.e., Model 3) included the main effects of age, gender, and the quality of the parent-adolescent relationship, a random intercept, and the random slope of age. Descriptive results can be found in Table 1 and a description of the results of the final model is presented in Table 2. The results of the final model showed a significant, negative main effect of the quality of the parent-adolescent relationship on initial trust, indicating that higher quality parent-adolescent relationships are associated with less initial in strangers (see Figure 3 and Table 2). During the model-building procedure, the random slope of the quality of the parent-adolescent relationship, the two-way interaction between age and the quality of the parent-adolescent relationship, and the three-way interaction between age, the quality of the parent-adolescent relationship, and gender appeared to be nonsignificant and were thus not included in the final model. The results of the log-likelihood ratio test for the comparison between the final model, i.e., Model 3, and the previous best-fitting model, i.e., Model 2, were χ^2^ (1) = 12.53, *p* < .001 (AIC Model 3: 6170; AIC Model 2: 6181; BIC Model 3: 6213; BIC Model 2: 6218). The explained variance of the final model was .47, which means that this model (including both the fixed and random effects parts) explained 47% of the variance in trust behavior.

**Table 1.** Descriptive Results per Wave

	Wave 1			Wave 3			Wave 5		
	*M* (*SD*)	Min	Max	*M* (*SD*)	Min	Max	*M* (*SD*)	Min	Max
Parent relationship	66.1 (11)	31	89	67.1 (15.4)	16	89	63.9 (15.5)	14	89
Peer relationship	55 (13.6)	9	82	51.7 (12.9)	11	76	51.4 (12.5)	10	77
Trust behavior	3.2 (1.9)	0	10	3.6 (2.2)	0	10	4.1 (2.5)	0	10

*Note.* Scores on trust behavior could range from 0 to 10. Scores on the quality of parent-adolescent and peer-adolescent relationship could range from −11 to 89. Min refers to the minimally attained value. Max refers to the maximally attained value.

**Table 2.** Frequentist Analyses of the Final Model Using the Quality of the Parent-Adolescent Relationship as a Predictor of Trust Behavior

	*B*	Standard deviation/Standard error	*t*-value (*p*-value)	95% CI^a^	
				Lower	Upper
Random effects					
Intercept participant		1.27		0.48	1.82
Slope age		0.59		0.32	0.80
Residual		1.60		1.48	1.68
Fixed effects					
Intercept	4.15	0.33	12.52 (<.001)	3.53	4.78
Age	0.39	0.06	6.54 (<.001)	0.27	0.52
Gender	−0.85	0.13	−6.33 (<.001)	−1.15	−0.60
Parent relationship	−0.02	0.004	−3.55 (<.001)	−0.02	−0.007

*Note.* The results of the fit of the final model reporting beta coefficients, standard deviations of the random effects, standard errors of the fixed effects, *t*-values, *p*-values, and the 95% confidence intervals (CI). Boys are coded as 0, girls are coded as 1.

^a^The 95% CI of the random effect is on the standard deviation of the effect as the *lmerTest* package does not report beta coefficients and *p*-values for random effects. The 95% CI of the fixed effect is on the beta coefficient.

### The Quality of the Peer-Adolescent Relationship

The model-building procedure that concerned the association between the peer-adolescent relationship and trust showed that the final model (i.e., Model 2) included the main effects of age and gender, a random intercept, and the random slope of age. A description of the results of the final model is presented in Table 3. During the model-building procedure, the random slope of the quality of the peer-adolescent relationship, the main effect of the quality of the peer-adolescent relationship, the two-way interaction between age and the quality of the peer-adolescent relationship, and the three-way interaction between age, the quality of the peer-adolescent relationship, and gender appeared to be nonsignificant and were thus not included in the final model. The results of the log-likelihood ratio test for the comparison between the final model, i.e., Model 2, and the previous best-fitting model, i.e., Model 1, were χ^2^ (2) = 31.55, *p* < .001 (AIC Model 2: 6318; AIC Model 1: 6345; BIC Model 2: 6355; BIC Model 1: 6372). The explained variance of the final model was .46, which means that this model (including both the fixed and random effects parts) explained 46% of the variance in trust behavior.

**Table 3.** Frequentist Analyses of the Final Model Using the Quality of the Peer-Adolescent Relationship as a Predictor of Trust Behavior

	*B*	Standard deviation/Standard error	*t*-value (*p*-value)	95% CI^a^	
				Lower	Upper
Random effects					
Intercept participant		1.27		0.46	1.79
Slope age		0.60		0.37	0.80
Residual		1.60		1.49	1.70
Fixed effects					
Intercept	3.11	0.15	20.24 (<.001)	2.79	3.41
Age	0.40	0.06	6.87 (<.001)	0.30	0.52
Gender	−0.82	0.13	−6.22 (<.001)	−1.09	−0.55

*Note.* The results of the fit of the final model reporting beta coefficients, standard deviations of the random effects, standard errors of the fixed effects, *t*-values, *p*-values, and the 95% confidence intervals (CI). Boys are coded as 0, girls are coded as 1.

^a^The 95% CI of the random effect is on the standard deviation of the effect as the *lmerTest* package does not report beta coefficients and *p*-values for random effects. The 95% CI of the fixed effect is on the beta coefficient.

### Post-Hoc Bayesian Analyses

The results of the above-reported models indicated no statistical support for the hypothesized relationships between the quality of parent-adolescent and peer-adolescent relationships as predictors of trust behavior. We only found an unexpected negative association between the quality of parent-adolescent relationship and trust behavior. Using frequentist analysis as done in the above-reported models, it is only possible to find evidence in support of the alternative hypothesis (H_1_) but not in favor of the null hypothesis (H_0_) (Dienes, 2014). Therefore, post-hoc Bayesian analyses were employed to quantify the degree of evidence for the H_0_, the H_1_, or determine that the result is inconclusive (Kruschke & Liddell, 2018), meaning there is no more support for either the H_0_ or the H_1_ (Wagenmakers et al., 2018). The post-hoc Bayesian analyses were not planned a priori, hence these analyses were not described in the preregistration.

We applied the default priors, assuming that model parameters could vary between a theoretical minimum and maximum (Klauer et al., 2024), and we used the Bayes factor (BF) to quantify the degree of evidence for either the H_0_ or the H_1_. We used the following rules of thumb to interpret the BF: values of BF_10_ higher than 100 provide decisive evidence for H_1_, 30-100 very strong evidence for H_1_, 10-30 strong evidence for H_1_, 3-10 substantial evidence for H_1_, 1-3 trivial evidence for H_1_, whereas values 0.33-1 indicate trivial evidence for H_0_, 0.10-0.33 substantial evidence for H_0_, 0.03-0.10 strong evidence for H_0_, 0.01-0.03 very strong evidence for H_0_, and values lower than 0.01 decisive evidence for H_0_ (Jeffreys, 1996).

Similar to the model-building procedures that were used for the frequentist analyses, two multilevel modeling procedures were set up for the Bayesian analyses. Both procedures consisted of the same six steps as in the frequentist analyses and followed a general recommendation for Bayesian model comparison presented by Kruschke and Liddell (2018). Analyses were performed in R version 4.3.1 (RCoreTeam, 2020) using the *brms* package (Bürkner, 2018).

The results for the modeling procedure when the quality of the parent-adolescent relationship was used as a predictor indicated the best overall model fit for Model 6. Table 4 presents the results of the model comparisons, and Table 5 presents the results of the Bayesian hypothesis and post-hoc testing for Model 6. First, trivial evidence was found for the H_1_ when the interaction between age and the quality of the parent-adolescent relationship on trust behavior was examined. Second, concerning the hypothesized positive association between the quality of the parent-adolescent relationship and trust behavior (independent of age), the results indicated there was substantial evidence for the H_0_. Please note that the rules of thumb to interpret BF indicate that substantial evidence for H_0_ concerns weak evidence. Given that this result indicated an unexpected negative estimate for the relationship between the quality of the parent-adolescent relationship and trust (see Table 5), we post-hoc examined the BF_10_ for the reversed effect, i.e., a negative association between the quality of the parent-adolescent relationship and trust behavior, independent of age. Also here, the results indicated substantial evidence for H_1_ when examining this reversed effect. Last, trivial evidence was found for the exploratory three-way interaction between age, the quality of the parent-adolescent relationship, and gender (in both directions). Given that trivial evidence was found for the interaction effects in both directions, possible simple slopes were not inspected. In sum, post-hoc Bayesian analyses with the quality of the parent-adolescent relationship as a predictor showed only trivial and weak evidence for the association between the quality of the parent-adolescent relationship and trust, with and without interaction effects of age and gender.

**Table 4.** Bayesian Analyses of the Model Comparisons Using the Quality of the Parent-Adolescent Relationship as a Predictor of Trust Behavior

Model	ELPD difference (standard error)^a^	*R*^2^ [95% CI]
Null model	−44.9 (9.3)	
Model 1	−184.4 (15.8)	.07 [.05, .10]
Model 2	−3.6 (2.9)	.02 [.01, .03]
Model 3	−2.0 (1.6)	.02 [.01, .04]
Model 4^b^	0.0 (1.5)	.02 [.01, .04]
Model 5	−14.4 (3.8)	.02 [.01, .04]
Model 6		.04 [.02, .05]

^a^ELPD difference is the discrepancy between the expected log predicted density measures when comparing two consecutive models, e.g., the comparison between the null model and Model 1 resulted in an ELPD difference of −44.9, indicating that the null model has a worse fit than Model 1.

^b^As the ELPD difference between Model 4 and Model 5 was 0.00, an additional comparison was made between Model 4 and Model 6, resulting in an ELPD difference of −14.4 (3.9).

**Table 5.** Bayesian Hypothesis and Post-hoc Testing Using the Quality of the Parent-Adolescent Relationship as a Predictor of Trust Behavior (Model 6)

Hypothesis	β [95% CI]	*SE*	BF_10_	Evidence
Age >0	0.26 [–0.20, −0.74]	.28	4.54	Substantial for H_1_
Gender <0	−0.16 [–1.33, 1.04]	.72	1.45	Trivial for H_1_
Parent relationship > 0^a^	−0.01 [–0.03, 0.01]	.01	0.14	Substantial for H_0_
Parent relationship <0	−0.01 [–0.03, 0.01]	.01	6.91	Substantial for H_1_
Age*parent relationship <0	<0.01 [–0.01, 0.01]	<.01	2.26	Trivial for H_1_
Age*parent relationship*gender > 0^b^	<0.01 [–0.01, 0.01]	<.01	1.17	Trivial for H_1_
Age*parent relationship*gender < 0^b^	<0.01 [–0.01, 0.01]	<.01	0.86	Trivial for H_0_

^a^This effect tests the hypothesis that the quality of the parent-adolescent relationship and trust behavior are positively associated. However, given that we found an unexpected negative β for this relationship, we post-hoc examined the BF_10_ for the reversed effect (i.e., parent relationship <0).

^b^The three-way interaction between age, the quality of the parent-adolescent relationship, and gender was tested exploratory, and, therefore, this effect was tested in both directions (i.e., age*parent relationship*gender >0 and age*parent relationship*gender <0).

The results of the modeling procedure with the quality of the peer-adolescent relationship as a predictor indicated the best overall model fit for Model 6. Table 6 presents the results of the model comparisons, and Table 7 presents the results of the Bayesian hypothesis and post-hoc testing for Model 6. First, trivial evidence was found for the H_1_ when the interaction between age and the quality of the peer-adolescent relationship on trust behavior was examined. Second, concerning the association between the quality of the peer-adolescent relationship and trust behavior (independent of age), the results indicated trivial evidence for the H_1_. Last, trivial evidence was found for the exploratory three-way interaction between age, the quality of the peer-adolescent relationship, and gender (in both directions) and possible simple slopes were therefore not inspected. In sum, post-hoc Bayesian analyses with the quality of the peer-adolescent relationship as a predictor showed only trivial evidence for the positive association between the quality of the peer-adolescent relationship and trust, with and without interaction effects of age and gender.

**Table 6.** Bayesian Analyses of the Model Comparisons Using the Quality of the Peer-Adolescent Relationship as a Predictor of Trust Behavior

Model	ELPD difference (standard error)^a^	*R*^2^ [95% CI]
Null model	−45.5 (9.2)	
Model 1	−16.4 (7.9)	.07 [.05, .10]
Model 2	−0.3 (2.4)	.06 [.04, .09]
Model 3	−180.5 (16.9)	.06 [.04, .09]
Model 4	−165.1 (18.3)	.02 [.01, .03]
Model 5	−177.3 (17.7)	.07 [.04, .09]
Model 6		.03 [.02, .05]

^a^ELPD difference is the discrepancy between the expected log predicted density measures when comparing two consecutive models, e.g., the comparison between the null model and Model 1 resulted in an ELPD difference of −45.5, indicating that the null model has a worse fit compared to Model 1.

**Table 7.** Bayesian Hypothesis and Post-hoc Testing Using the Quality of the Peer-Adolescent Relationship as a Predictor (Model 6)

Hypothesis	β [95% CI]	*SE*	BF_10_	Evidence
Age >0	0.43 [0.31,0.54]	.07	Inf	Decisive for H_1_
Gender <0	0.27 [–0.04,0.57]	.17	0.08	Strong for H_0_
Peer relationship >0	<0.01 [–0.01, 0.01]	<.01	1.35	Trivial for H_1_
Age*peer relationship >0	<0.01 [–0.01, 0.01]	<.01	1.35	Trivial for H_1_
Age*peer relationship*gender > 0^a^	<0.01 [–0.01, 0.01]	<.01	1.46	Trivial for H_1_
Age*peer relationship*gender <0	<0.01 [–0.01, 0.01]	<.01	0.68	Trivial for H_0_

^a^The three-way interaction between age, the quality of the peer-adolescent relationship, and gender was tested exploratory and, therefore, this effect was tested in both directions (i.e., age*peer relationship*gender >0 and age*peer relationship*gender <0).

## Discussion

Including three waves of data collection across adolescence, we separately examined the association between the quality of the parent-adolescent relationship and trust in strangers and the peer-adolescent relationship and trust in strangers. Contrary to our hypothesis, the results indicated no evidence of developmental changes regarding these two associations. Independent of age, we unexpectedly found that higher quality relationships with parents were related to lower levels of trust in strangers, while no evidence was found for an association between the quality of peer-adolescent relationships and trust in strangers (independent of age). The exploratory comparison of associations by gender, revealed no evidence for differences between boys and girls.

Concerning developmental changes, the results of the current study indicated no evidence for developmental changes regarding the association between the quality of the parent-adolescent relationship, the quality of the peer-adolescent relationship, and trust behavior, even though previous research by our group (Sijtsma et al., 2023) indicated that, in this sample, trust behavior alone did increase with age. In the presence of a large sample size, it is hard to speculate about explanations for the non-significant findings. The results of the Bayesian analyses do strengthen the idea that there is trivial (i.e., meaningless) evidence for development with regard to the association between the quality of parent-adolescent relationships, peer-adolescent relationships, and trust. This is in line with the findings of Stolle and Nishikawa (2011) who found that it is unlikely that parents serve as role models of trust behavior for children of 12 years old (however, do note that they used a different measure of trust behavior namely the Comparative Children’s Survey on the political and social role that families and schools may have in developing trust in strangers).

Contrary to our hypothesis, the results indicated that, independent of age, higher-quality relationships with parents were related to lower levels of trust behavior. This finding could be a false positive finding (i.e., a Type-1 error) or in fact a true finding; however, it is difficult to speculate about possible explanations. It might be that adolescents who experience low-quality parent-adolescent relationships are more motivated to establish relationships with strangers and, therefore, more inclined to trust others. For example, it has been shown that children with an insecure attachment relationship with their mother may, in certain circumstances, rely less on information provided by their mother and be more inclined to accept information provided by a stranger (Corriveau et al., 2009). However, it should be noted that the additional Bayesian analyses indicated that the evidence for the negative association between the quality of the parent-adolescent relationships and trust in strangers is only weak. Also, we would like to highlight that this post-hoc interpretation is only speculative and future research should replicate this finding before any conclusions are drawn. Another suggestion for future research is to collect more information about the parent-adolescent relationship, for example, by interviewing adolescents and parents on their views on their parent-adolescent relationship.

Furthermore, in contrast to our hypotheses, the results of the current study indicated that, independent of age, no evidence was found for an association between the quality of the peer-adolescent relationships and trust behavior. In line, Bayesian analyses showed only trivial (i.e., meaningless) evidence for this relationship. Based on the attachment theory, it has been proposed that the relationship and interactions between children and their close others can serve as a basis for how social situations with strangers are approached (Bowlby, 1969, 1973, 1980). This idea has been applied to trust interactions specifically, as one can use trust experiences from interactions with close others and generalize these experiences to trust interactions with strangers (Stolle, 2002). Our findings are in contrast to the results of a recent study by Jambon and Malti (2022) which indicated that, for children, the quality of peer-adolescent relationships and having trust beliefs in others are positively associated. As they point out, they used a questionnaire to measure trust which may be informative on broad and global trust beliefs (for example, “I think most people are fair.”; “I trust other people.”; “I believe most people can be trusted.”). This measure of trust may be seen as a different kind of trust than the type that is measured using the Trust Game. The measure of trust used in the current study taps into a form of trust building during a social interaction, which includes a trust target (i.e., the partner) and consequences related to one’s trust decisions (i.e., the outcomes for both the participant and the partner at the end of a trial and game). Combined, our findings and the results by Jambon and Malti (2022) might suggest that the quality of the interpersonal relationships with close others is not related to measures of trust that are manifested in behavioral tasks, but significant associations have been found when general trust beliefs were measured using questionnaires.

We further explored if the examined associations differed by gender, but the results indicated no evidence for differences between boys and girls. We did not formulate hypotheses for possible gender differences because previous studies showed contrasting results regarding gender differences and social-cognitive functions involved in trust behavior. For example, a significant association between the quality of peer-adolescent relationships and ToM skills were found in boys (Białecka-Pikul et al., 2021), while no significant gender differences were found for the association between the quality of close relationships and empathy (Boele et al., 2019) and the quality of peer-adolescent relationships and trust (Jambon & Malti, 2022). The results of a meta-analysis in adults did show that males have higher levels of initial trust in strangers than females (Van den Akker et al., 2020). Future studies may extend the period of data collection to span a longer period of adolescence to examine whether gender differences emerge in later stages of adolescence or young adulthood.

The results of the current study should be seen in light of several considerations. First, the Trust Game was played with a hypothetical partner and not with a human counterpart. This type of partner was necessary as we used a longitudinal design where deception was undesirable and unfeasible. Nonetheless, multiple studies show that playing games with computer counterparts elicits similar (though weaker) responses compared to playing with human counterparts (Kircher et al., 2009; Rilling et al., 2004; Van ’t Wout et al., 2006). This is especially the case when the computer’s behavior is adaptive to the participant’s behavior, as is the case with the algorithm we used to simulate the cartoon’s behavior. Second, participants were instructed that they would receive questions about their parents and themselves, and asked to indicate to what extent the questions applied to them. After completing the questionnaires, participants were not asked to specify which parent or caregiver they had in mind, which could also include primary caregivers other than their parents (e.g., grandparents, foster parents, or youth care parents). It is also possible that participants filled out the questionnaire with multiple caregivers in mind, potentially explaining the low Cronbach’s alpha value for the IPPA-R Parent at Wave 1 (i.e., .67). For future studies, it is advisable to verify which caregiver participants had in mind in order to have a clear understanding of the measure. Also, to acquire a more nuanced and differentiated view, future research may distinguish between multiple caregivers, as the quality may vary for different caregivers (see for example Keizer et al. (2019) and Lu et al. (2022)). Last, the sample used in the current study was not diverse, as it mainly consisted of Western adolescents who are in the top 40% of Dutch secondary education. This homogenous sample makes it difficult to generalize the findings to adolescents of different socioeconomic backgrounds. It is possible that the socioeconomic background of a family, for example, the extent to which families experience financial stress or whether adolescents need to contribute to their family’s financial situation, affects the relationship between parents and adolescents. Future studies should investigate these possible moderating effects.

To conclude, unexpectedly, no evidence was found for developmental changes regarding trust and both the quality of parent-adolescent and peer-adolescent relationships, while weak evidence was found for a negative relationship between the quality of parent-adolescent relationships and trust in strangers. With regard to how trust relationships with known others transfer to trust in strangers, future research could differentiate between relationships with different caregivers and various socioeconomic backgrounds and further investigate how the method to examine trust (e.g., behavioral tasks versus questionnaires) affects the possible association between trust and parent-adolescent and peer-adolescent relationships.

## Supplemental Material

Supplemental material - The Quality of Parent-Adolescent and Peer-Adolescent Relationships and Trust in Strangers Supplemental material for The Quality of Parent-Adolescent and Peer-Adolescent Relationships and Trust in Strangers by Hester Sijtsma, Mariët van Buuren, Jacek Buczny, Miriam Hollarek, Reubs J. Walsh, Nikki C. Lee, Lydia Krabbendam in The Journal of Early Adolescence

## Data Availability

The participants and their parents did not provide explicit consent for the public archiving of the research data; therefore, the data are not stored in a public repository. Anonymized data will be made available to individual researchers upon request when compatible with the General Data Protection Regulation. Additionally, researchers that request the data will be required to have obtained ethical approval from their host institution and are not allowed to share the data.*
