# Supplementary material for: The Quality of Parent-Adolescent and Peer-Adolescent Relationships and Trust in Strangers
Source: J Early Adolesc. 2025 Sep 23;46(7):1022–50. doi: 10.1177/02724316251379489 (PMC13258205; doi:10.1177/02724316251379489)
Supplement: Supplemental material - The Quality of Parent-Adolescent and Peer-Adolescent Relationships and Trust in Strangers [file sj-pdf-1-jea-10.1177_02724316251379489.pdf]

Supplementary Files

In the current analyses, trust was measured using the investment that the participant made during the first trial of the first condition that was administered. The investments made during the remaining trials and the trials of the second condition were not included in the analyses.

In each trial, the partner returned an amount of money to the participant. The amount was determined by a preprogrammed algorithm. The specific return was determined by the participant's investment multiplied by a predefined factor.

To establish an identical baseline of trustworthiness for both partners, the algorithm was equally trustworthy during the first five trials of both conditions. The factor for trial 1 was randomly selected from the values 1.2, 1.3, and 1.4. For trial 2 to trial 5, the factor increased by 0.1 (in steps of 0.1 with a minimum factor of 1.2 and a maximum factor of 1.4) in case the participant increased the investment compared to the investment during the previous trial. The factor remained constant in case the investment decreased or did not change compared to the previous trial.

From trial 6 onwards, the factor determining the partner's return was different for both conditions. For the trustworthy condition holds that the partner's trustworthiness increased compared to the first five trials. The factor for trial 6 was randomly chosen between 1.5 and 2.0 (in steps of 0.1). For trial 7 through trial 15, the factor increased by 0.1 in case the participant's investment increased compared to the investment during the previous trial (with a minimum factor of 1.5 and a maximum factor of 2.0). The factor remained constant in case the participant's investment decreased or did not change compared to the previous investment.

In contrast, in the untrustworthy condition, the partner's trustworthiness decreased compared to the first five trials. The factor for trial 6 was randomly chosen between 0.7 and 1.2 (in steps of 0.1). The factor for trial 7 through trial 15 decreased by 0.1 when the participant's investment increased compared to the previous investment (with a minimum

factor of 0.7 and a maximum factor of 1.2). The factor remained constant in case the participant's investment decreased or did not change compared to the previous investment. In sum, both algorithms were adaptive to increases in the participants' investments: in the trustworthy condition, the relative returns increased in response to increased investments, whereas in the untrustworthy condition, the relative returns decreased.
